# Personalized Medicine for Liver Disease: From Molecular Mechanisms to Potential Targeted Therapies

**DOI:** 10.3390/jpm12050663

**Published:** 2022-04-21

**Authors:** Aaron W. Bell

**Affiliations:** Department of Pathology, School of Medicine, University of Pittsburgh, Pittsburgh, PA 15261, USA; bellaaro@pitt.edu

This Special Issue, entitled “Personalized Medicine for Liver Disease: From Molecular Mechanisms to Potential Targeted Therapies”, includes 11 publications from colleagues working on various liver diseases including non-alcoholic fatty liver disease (NAFLD), alcoholic liver disease (ALD), hepatocellular carcinoma (HCC), primary biliary cholangitis (PBC), as well as various treatment modalities including pharmacotherapies and liver transplantation. 

Fatty liver disease (FLD) has been traditionally classified as either non-alcoholic fatty liver disease (NAFLD) or alcoholic liver disease (ALD). As critical inducers of hepatic steatosis, both high-fat diets and chronic alcohol consumption are profoundly associated with the recent burden of metabolic syndromes. In Fact, Kim et al. show that concomitant intake of dietary fat and alcohol can work synergistically to worsen liver injury [1]. With the global increase in obesity, type 2 diabetes, and other metabolic syndromes, NAFLD has become the leading cause of chronic liver disease, affecting 25% of adults worldwide. These patients are at the top of the list for liver transplantation. In addition, FLD is the most common cause of HCC in Western countries. Technical advances in genomic approaches have revealed that genetic factors also contribute to the risk of developing FLD and have led to the discovery of disease biomarkers, some of which, such as HSD17B13, may slow disease progression and are considered promising therapeutic targets [2]. 

Given the prevalence of NAFLD today, its heterogeneous presentation with multiple possible comorbidities, and the variety of therapeutic treatment options, it is critical to stratify patients and tailor treatments to each case. In this Special Issue, Finotti et al. provide a concise but detailed review of NAFLD pathogenesis and the pharmacological and surgical treatment options [3].

The importance of stratifying and tailoring treatments is further strengthened in a retrospective study looking at clinical lab parameters before and during ursodeoxycholic acid (UCDA) treatment of patients with primary biliary cholangitis (PBC) [4]. UDCA is the first-line pharmacotherapy for PBC and is well tolerated. Gazda et al. found that blood tests could predict which patients would respond well and which may require additional intervention with OCA and fibrate drugs [4].

Hepatocellular carcinoma (HCC) remains a major health problem worldwide with a continuously increasing prevalence. Despite the introduction of targeted therapies such as the multi-kinase inhibitor sorafenib, treatment outcomes are not encouraging. The prognosis of advanced HCC is still dismal, emphasizing the need for novel effective treatments. Apart from the various risk factors that predispose to the development of HCC, epigenetic factors also play functional roles in tumorigenesis. Histone deacetylases (HDAC) are enzymes that regulate critical cellular functions via acetylation of histone and non-histone protein targets. Considering that HDAC activity is elevated in HCC, treatment strategies with HDAC inhibitors showed some promise. Nevertheless, HCC is a prominent multifactorial disease with numerous etiologies making broad-spectrum chemotherapies and treatments unreliable, thereby necessitating precision therapies. Bioinformatics and multi-omic approaches that identify biomarkers and therapeutic targets such as stathmin-1, HGF, and miR-29a may be the key to realizing true therapeutic results with precision therapies in specific subclasses of HCC [5,6,7]. Wang et al. show that using the telomerase inhibitor MST-312 could attenuate viability and decrease migration and invasion capabilities of stathmin-1-high expression HCCs [6]. Crisper/CAS9 gene editing holds promise for precision therapeutic interventions to reprogram diseases cells. Lee et al. show evidence that through such intervention knockout of a key growth factor, HGF, in liver cancer cell lines can reduce proliferation and migration, and increase apoptosis of HCC cells [5].

microRNAs (miRNA) are known to widely participate in the regulation of expression of oncogenes and tumor suppressor genes, directly and epigenetically, and play a major role in cancer progression. Regarding HCC, miR-29a is believed to be hepatoprotective, and Yang et al. give a detailed review of its molecular mechanisms and the diagnostic and prognostic value of its expression and activity [7]. 

Liver transplantation has become much more commonplace as a treatment option for a variety of liver diseases including non-alcoholic fatty liver disease and hepatocellular carcinoma, but the procedure is fraught with difficulties due to a shortage of transplantable organs, the need for immunosuppressive drugs, and post-surgical complications within the liver and other organ systems, all of which necessitate identifying the best patients to transplant. 

The high demand for liver transplantation has led to the development of living-donor partial liver transplants, as well as the transplantation of isolated liver cells (hepatocytes). Cell transplantation requires the isolation of hepatocytes from an intact organ and subsequent purification steps, which are subject to the risk of contamination and increased damage due to ischemia. Chen et al. evaluate the use of a semi-automated closed-circuit system “Cell Saver Elite” in isolation of transplantable cells [8]. Although their study was limited to rodent livers, they show a proof of concept that automated cell recovery can generate viable, functional, and transplantable cells, comparable to traditional methods in less time and with less risk of contamination. This could be of great use to advance the field and help to alleviate the need for whole or even partial organs for transplant. 

Nevertheless, the shortage of transplantable organs makes identifying the patients most in need of a life-saving transplant critical. Acute chronic liver failure can arise from a variety of confounding insults in patients with underlying cirrhosis. The heterogeneity in etiology leads to variable disease courses and has hampered accurate diagnosis and prognosis. Lu et al. investigate dynamic changes in clinical markers, especially within the first 2 weeks of clinical admission, that aid in predicting poor prognosis and indicate the necessitation of fast-tracking therapeutic interventions or transplant prioritization [9].

Immunosuppression is required post-liver transplantation to ensure patient and graft survival, and tacrolimus is the most widely used immunosuppressant drug post-transplant. However, blood concentrations post-transplant must be closely monitored to minimize toxic effects and infections. Hsiao et al. reveal that long-term survival post-transplant was optimum when tacrolimus levels were at or above 4.6 ng/mL [10]. 

Post-liver transplant acute kidney injury is a prevalent complication that can lead to chronic kidney injury and even death. Several risk factors have been associated including high BMI, low albumin levels, poor graft quality, prior kidney dysfunction, and inflammation. Antithrombin III has anticoagulation and anti-inflammatory properties and in a retrospective study, Kim et al. identify ATIII levels as a predictive factor in the incidence of post-transplant acute kidney injury [11]. 

Liver diseases such as NAFLD, ALD, HCC, and PBC may be lumped into a classification of a particular disease, but each individual case is in and of itself unique and therefore must be treated as such. The future of medicine is tailored, targeted, precision therapeutics personalized for each patient.
